# Tailoring Tomato (*Solanum lycopersicum* L.) Traits to Microclimates: A Multilocation Evaluation of Yield and Quality Responses in Western Ethiopia

**DOI:** 10.1155/sci5/6345142

**Published:** 2025-04-28

**Authors:** Usman Mohammed Ali, Desalegn Negasa Soresa, Tilahun Wondimu Fufa

**Affiliations:** ^1^Department of Plant Sciences, Faculty of Agriculture, Wollega University, Shambu, Oromia, Ethiopia; ^2^Bako Agricultural Research Center, Oromia Agricultural Research Institute, Bako, Oromia, Ethiopia

**Keywords:** fruit quality, genotype–environment interaction, microclimatic adaptation, off-season production, tomato cultivars, yield optimization

## Abstract

Tomato (*Solanum lycopersicum* L.) is a crucial crop for food security and income generation in Western Ethiopia. However, unsuitable cultivar choices and misalignment between genotype and microclimate conditions often constrain its productivity. This study aimed to evaluate the performance of eight national tomato cultivars and one local landrace across diverse microclimates in Western Ethiopia during the 2022/2023 off-season cropping period. A randomized complete block design with three replications was employed to assess growth, yield, and quality parameters at two locations: Bako Tibe and Gambella Tare. The results revealed significant genotype–environment interactions affecting various traits, underscoring the necessity for microclimatic adaptation in tomato cultivation. Cochoro emerged as a high-yielding cultivar, achieving marketable fruit yields of 91.5 t/ha at Bako and 84.28 t/ha at Gambella Tare, while Komto (43 t/ha at Bako Tibe) and Geli-Lema (42.2 t/ha at Gambella Tare) exhibited the lowest yields. Melka-Salsa demonstrated superior fruit quality, particularly in ascorbic acid content (26.30 mg/100 g), while Cochoro had the lowest (12.81 mg/100 g). A strong positive correlation (*r* = 0.998^∗∗^) was identified between total marketable fruit yield and fruit weight per plant, suggesting potential targets for future breeding efforts. This study highlights the critical role of genotype–environment interactions in optimizing tomato production across microclimates in Western Ethiopia. These findings provide valuable information for farmers and stakeholders to select the most suitable cultivars, enhancing yields and improving farmer incomes. Future research should prioritize expanded genotype–environment interaction studies, breeding programs targeting yield components and stress resilience traits, and the development of location-specific agronomic packages integrating optimized irrigation, nutrient management, and climate-smart pest control strategies.

## 1. Introduction

Tomato (*Solanum Lycopersicum* L.) is one of the world's most important and popular fruit vegetables. It is a self-pollinated annual crop and belongs to the family Solanaceae with chromosome number 2*n* = 2*x* = 24 [1]. It is reported as the most popular vegetable crop grown in home gardens and the second most consumed after potato (*Solanum tuberosum* L.) worldwide [2].

Tomatoes, botanically classified as berries, transcend their categorization as vegetables by offering a treasure trove of health benefits. Rich in vitamins, minerals, dietary fibers, and antioxidants like lycopene, they contribute significantly to balanced diets and disease prevention [3, 4]. Cultivated globally in diverse settings like open fields, greenhouses, and screen houses; tomatoes hold immense economic importance [2, 5, 6].

Temperature plays a critical role in tomato growth and development [7]. Optimal temperatures for tomato cultivation generally range between 20°C and 25°C during the day and 15°C to 20°C at night. Deviations from these ranges can lead to physiological stress, affecting flowering, fruit set, and overall yield [8]. For instance, high temperatures above 30°C can cause blossom drop and reduce fruit quality due to increased respiration rates and water loss [9].

Rainfall patterns significantly influence the performance of different tomato cultivars. Consistent with previous research [10], excessive rainfall during critical growth stages, such as flowering and fruit set, negatively impacted yield and fruit quality. This is likely due to increased disease incidence, nutrient leaching, and impaired pollination [11]. Conversely, insufficient rainfall resulted in water stress, leading to reduced plant growth, delayed maturity, and smaller fruit size [12]. Cultivars with drought-tolerant traits, such as deep root systems and efficient water use, exhibited better performance under water-limited conditions [13].

Altitude has a significant influence on the performance of different tomato variety [14]. Previous research [15] has shown that higher altitudes are associated with lower temperatures, higher UV radiation, and lower atmospheric pressure, all of which can impact plant growth, development, and yield. In our study, cultivars adapted to higher altitudes exhibited better performance at higher elevations, characterized by increased fruit yield, improved fruit quality, and enhanced resistance to biotic and abiotic stresses.

In 2023, global tomato production reached a staggering 189 million tons, with China, India, Turkey, and the United States leading the pack [2]. Ethiopia, cultivating tomatoes since the 1930s, positions them as a crucial cash crop and source of food security [16, 17]. Despite widespread cultivation by smallholder farmers and commercial entities, Ethiopia's national yield (6.52–24 Mt ha^−1^) lags far behind the global average (34 Mt ha^−1^) and pales in comparison with major producers [2].

This yield disparity not only threatens national food security but also undermines the livelihoods of Ethiopian tomato farmers, particularly in Western Oromia. Limited access to improved cultivars adapted to Ethiopia's diverse agroecological landscapes emerges as a key culprit [18]. Coupled with inadequate varietal information and suboptimal agricultural practices, this lack of suitable cultivars exacerbates the yield gap between potential and reality [19, 20].

While previous research has explored tomato performance across diverse environments [21–25], studies specifically examining the impact of microclimatic variations on cultivar performance within Ethiopia remain limited. This study aims to address this knowledge gap by conducting a multilocation trial to evaluate cultivar performance across distinct microclimates; assessing a diverse germplasm, including both national cultivars and a local landrace; and comprehensively evaluating key agronomic traits beyond yield, such as growth and fruit quality. These combined approaches will provide valuable insights into the adaptability of different tomato genotypes to specific microclimatic conditions within the Ethiopian context.

### 1.1. Research Objectives

This study aims to bridge this crucial gap by evaluating the performance of selected tomato varieties in Bako Tibe and Gambella Tare, Western Oromia. By assessing their growth, yield, and quality parameters under these specific off-season conditions, the research seeks to:

• Evaluate agromorphological and quality variability among the selected tomato varieties.
• Investigate the relationship between tomato variety characteristics and their performance in the study locations.

### 1.2. Significance of the Study

The findings of this research will empower tomato producers in Western Oromia by providing valuable insights into cultivar performance under local off-season conditions. This knowledge will equip them to make informed choices when selecting tomato varieties, ultimately optimizing yield, quality, marketability, and farmer livelihoods. Moreover, the study contributes to bridging the gap in accessible varietal information, aiding researchers in developing improved cultivars. This research holds immense potential to bolster Ethiopia's tomato production, enhance food security, and empower its agricultural sector by addressing these critical knowledge gaps in specific Ethiopian contexts. By addressing these critical knowledge gaps, this research holds immense potential to bolster Ethiopia's tomato production, enhance food security, and empower its agricultural sector.

## 2. Materials and Methods

### 2.1. Description of the Experimental Area

**Figure 1:**
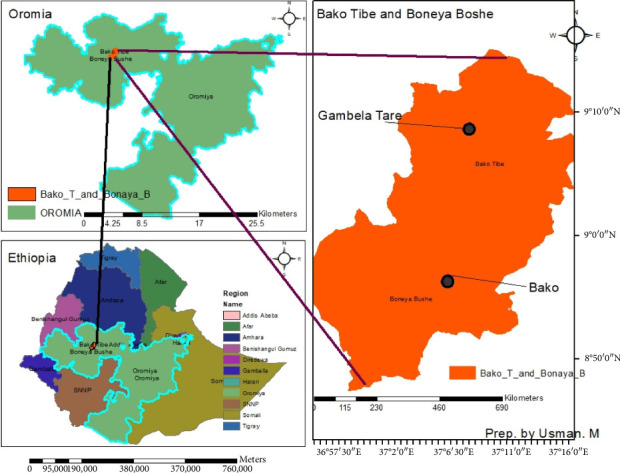
Map of the study sites in Gambella Tare and Bako Tibe districts, Western Ethiopia (Source: own sketch using ArcGIS).

The study was carried out at two locations, Bako Tibe and Gambella Tare, Oromia, Ethiopia, during the 2022–2023 cropping season. Bako Tibe is located 238.7 km west of Addis Ababa, situated between 8°55′18″ and 9°14′13″N latitude and 37°01′54″ and 37°17′07″E longitude (Figure 1). It has an elevation of 1560 m.a.s.l. and a subhumid agroecology with unimodal rainfall patterns, resulting in an annual mean rainfall of 1289 mm. The soil exhibits a reddish-brown color [26].

Gambella Tare, located 287 km west of Finfinne, is situated within the Ninth Agri-Business plc in the Bonaya Boshe District of East Wallaga. It has an elevation of 2697 m above sea level, with latitude of 9°4′0″N and a longitude of 37°01′0″E (Figure 1). The site comprises deep alluvial soil with a loam, clay loam, and silt loam texture. Gambella Tare experiences brief rains from March to April and long rains between June and September, with a moderate annual rainfall of around 1800 mm and a mean minimum and maximum temperature of 18°C and 27°C, respectively.

### 2.2. Justification for Site Selection

Bako Tibe and Gambella Tare were selected as experimental sites due to their contrasting microclimates within Western Ethiopia. Bako Tibe, with its lower altitude and subhumid agroecology, offers a distinct microclimate compared to the higher altitude and different rainfall patterns of Gambella Tare (Figure 1). This contrasting environment enables the evaluation of cultivar performance across a broader range of microclimatic conditions prevalent in Western Ethiopia. By assessing cultivar responses to these contrasting environments, the study provides valuable insights into genotype-by-environment interactions and facilitates the identification of cultivars adapted to specific agroecological zones. This strategic selection enhances the relevance and applicability of research outcomes to local farming practices and agricultural policies to improve tomato production in the area.

### 2.3. Experimental Design and Treatments

A randomized complete block design (RCBD) with three replications was employed at both locations. Eight tomato varieties and one local check (LC) (Table 1) were evaluated. Each plot size measured 3 m × 4 m, with spacing of 1 m between rows and 0.5 m within rows.

### 2.4. Experimental Design and Agronomic Practices

The studies were carried out under irrigation conditions during the off-season (October 2022–March 2023). At both sites, nursery beds measuring 1 m × 10 m in width and length were meticulously prepared, with a 5-cm elevation above the soil surface to ensure adequate drainage and prevent waterlogging. Prior to seeding, the nursery beds were preirrigated 2 days in advance to facilitate planting. The seeds were sown in rows spaced 20 cm apart, maintaining a sowing depth of approximately 0.5–1 cm. After covering the seeds with finely pulverized soil, the beds were lightly firmed and irrigated regularly until germination was complete. To optimize plant growth and development, seedlings were thinned to an intrarow spacing of 3 cm. After 35 days, vigorous and healthy seedlings were carefully transplanted to the prepared experimental field.

**Figure 2:**
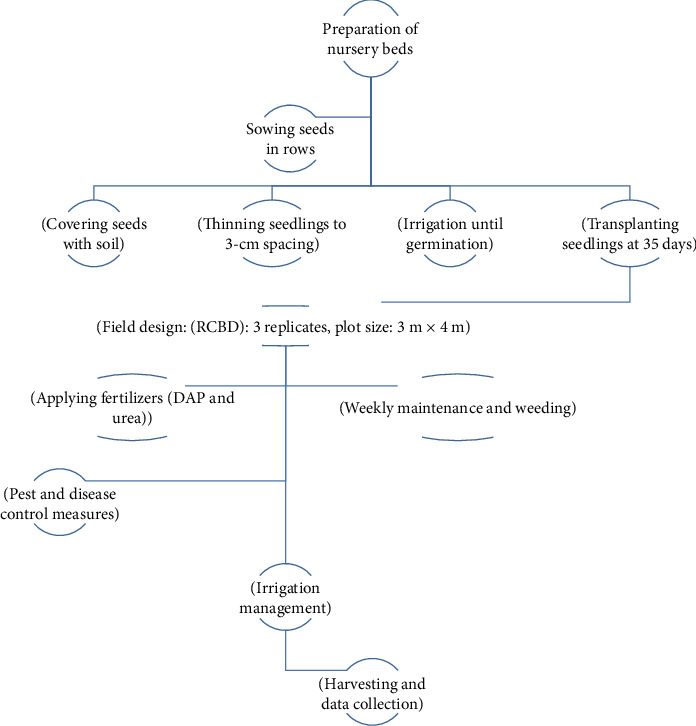
Tomato production workflow. Source: own sketch.

Nine tomato varieties were arranged in a RCBD with three replications. The plot size was 3 m × 4 m (12 m^2^). Transplanting of seedlings on the experimental field was done at the 3–5 true leaf stage when seedlings attained a height of about 23 cm (35 days after planting) (Figure 2).

The entire recommended dose of diammonium phosphate (DAP) (242 kg/ha^−1^) was applied during transplanting, while the recommended rate of urea (100 kg/ha^−1^) was split into three equal applications. The first one-third of urea was applied at transplanting, followed by the second one-third 21 days later. The final one-third was applied pentad after the second application [28].

The experimental field was meticulously tended by trained agricultural professionals who cultivated it weekly to ensure the tomatoes reached their peak ripeness. Stacking was carried out with great uniformity, and the weeds were diligently removed by hand up to four times throughout the season. Cupricide 77% WP a chemical, a highly effective bactericide, was used to ward off bacterial diseases such as bacterial wilt, and bacterial spots that can impact tomato plants. Furthermore, natural and synthetic pesticides and insecticides like ecogreen, Tutan, Helerat, Karate, and Mala were ingeniously incorporated to control the infestation of pests like spider mites, thrips, leaf miners, aphids, whiteflies, and leafhoppers. To prevent fungal diseases such as Alternaria, early and late blight, gray mold, and powdery mildew from presenting themselves, the crop was treated with the broad-spectrum fungicides Mancozeb and Bravo in the field. Finally, the ecogarden and agrofeed plant food and fertilizer are utilized to give the tomato plants essential nutrients that promote healthy growth and increase crop yield (Figure 2).

For the first 2 weeks, the experimental field was irrigated daily until the tomato crop was established uniformly and then within 3 days.

### 2.5. Data Collection

Data on a comprehensive set of 35 agronomic and quality traits were meticulously collected from ten randomly selected plants within each genotype at both experimental sites. Standardized protocols outlined in the “Descriptors for Tomato” by IPGRI [29] were strictly adhered to ensure data consistency and reliability (Table 2).

#### 2.5.1. Quantitative Traits

Quantitative traits are provided in Table 2.

The study evaluated 16 quantitative traits related to plant growth, yield, and fruit characteristics in the experimental plots. These traits included days to 50% flowering (DF), number of days to maturity (NDM), plant height (PH), number of primary branches per plant (NPBP), number of fruits per plant (NFSP), average fruit weight (AFW), number of clusters per plant (NCP), fruit pericarp thickness (FPTH), marketable fruit yield per plant (MFY), fruit size (FS), total fruit yield (TFY), fruit length (FL), number of locules (NL), fruits per cluster (FPC), fruit diameter (Fd), and stem thickness (STH). Measurements were taken following standardized protocols, including the use of vernier calipers, tape meters, and manual counting on randomly selected plants. Data collection methods were based on established references of IPGRI [29], ensuring consistency and accuracy in trait assessment (Table 2).

#### 2.5.2. Qualitative Traits

• Fruit morphology: fruit density and NL.
• Physicochemical properties: total soluble solids (TSS %), ascorbic acid content (%), and pH.

This comprehensive data collection approach aimed to capture a holistic picture of genotype performance across various aspects of growth, yield, and quality characteristics. This detailed information forms the foundation for subsequent analyses and interpretations of genotype–environment interactions within the study.

### 2.6. Statistical Analysis

Data from both experimental locations were subjected to separate and combined analysis of variance (ANOVA) using SAS 9.0 software [32]. Normality and equal variance test and transformation of data for some characters were done using Minitab software [33].

#### 2.6.1. Separate ANOVA

A RCBD model was employed for each location following Steel and Torrie [34]. Genotypes were considered fixed effects, and blocks were considered random effects. Mean comparisons were performed using Fisher's least significant difference (LSD) test at α = 0.05.

#### 2.6.2. Combined ANOVA

A combined ANOVA following Ntawuruhunga and Dixon [35] was conducted to assess genotype × location interactions. The RCBD model used included genotype, location, genotype × location interaction, and block effects.

#### 2.6.3. Justification for Statistical Techniques

The statistical analysis employed in this study utilized separate and combined ANOVAs through SAS 9.0 software, a standard approach in agricultural research for assessing treatment effects on crop performance. ANOVA is particularly effective for comparing multiple groups and determining significant differences among them while controlling for variability within treatments. The use of Minitab software for normality tests and data transformation before analyses was essential to meet the assumptions of ANOVA, normality, and homogeneity of variances, ensuring the validity of the results. Separate and combined analyses were conducted to evaluate cultivar performance within and across locations, respectively. This rigorous statistical framework not only enhances the reliability of the findings but also allows for robust interpretations regarding the influence of microclimatic factors on tomato traits. By employing these established statistical techniques, the study aligns with best practices in agricultural research methodology, facilitating credible conclusions that can inform future breeding and cultivation strategies.

#### 2.6.4. Correlation Analysis

Pearson correlation coefficients were calculated to identify significant relationships between yield and its components, as well as among yield components themselves [36]. This analysis aimed to explore potential associations between various traits and inform further interpretation of genotype performance.

## 3. Results and Discussion

### 3.1. Quantitative Traits and Performance Across Locations

The performance of nine tomato cultivars was assessed for yield and related quantitative traits under off-season conditions in two locations of Western Ethiopia. Significant genotypic variations were observed for all measured traits, indicating the potential for cultivar selection to improve production under these conditions. While the two-way ANOVA for location interaction revealed no significant differences for most traits (Table 3), exceptions were seen for days to flowering, days to maturity, and number of fruit clusters per plant. This suggests that environmental factors unique to each location may influence these specific aspects of plant development.

To further elucidate the impact of local conditions, separate ANOVAs were conducted for each location (Bako Tibe and Gambella Tare). This approach can aid in identifying the specific variables influencing tomato plant development, yield, and other traits in each environment [37, 38].

#### 3.1.1. DF

Significant genotypic variation (*p* ≤ 0.01) was observed for DF at both locations (Table 4). Melka-Shola exhibited the longest period (52.3 days), while Melka-Salsa flowered earliest (43.1 days) (Table 4). Variations linked to cultivar genetics, photoperiod, temperature, and potentially specific interactions. The findings align with Milkinesh and Negash [39] and highlight the influence of both genotype and environment on flowering timing.

#### 3.1.2. NDM

A highly significant (*p* < 0.01) difference across locations for days to maturity was observed in the study (Table 4). Variety-by-location interaction revealed differing maturity rankings between sites. ILu-Harar matured fastest (59.5 days), while LC took the longest (76.3 days) (Table 3). Early-maturing cultivars like ILu-Harar are potentially beneficial for early markets. Variations are attributed to environment, genetics, physiological factors, and fruit morphology. It aligns with Emami and Eivazi [40] and emphasizes the complexity of maturity timing.

#### 3.1.3. Number of Fruits per Plant

Highly significant (*p* < 0.01) genotypic differences were observed for the number of fruits per plant (Table 4). Melka-Salsa produced the most fruits (50.6), while the LC had the least (21.3). Variations are likely due to differences in cluster number, biotic factors, and temperature stress tolerance. This aligns with Fekadu et al. [41] and Chernet et al. [42], showing wide variation in fruit number. Temperature stress during reproductive stages could further influence fruit development.

#### 3.1.4. AFW (gm/Fruit)

**Figure 3:**
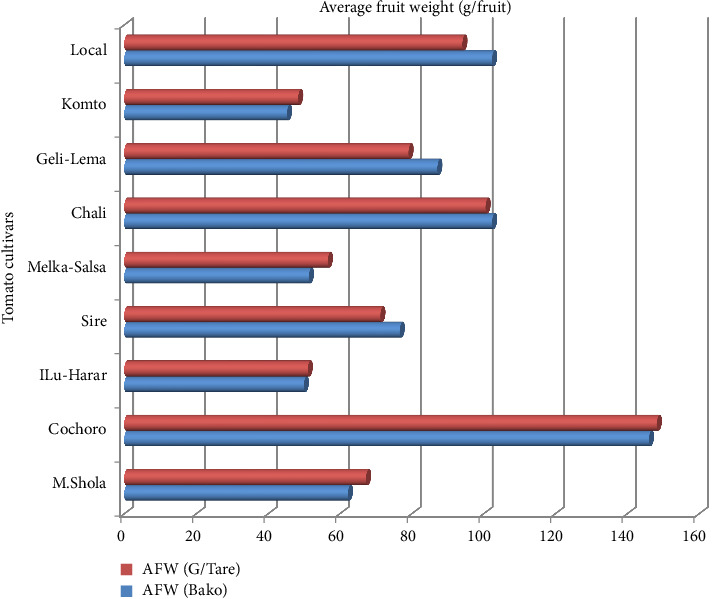
Comparison of average fruit weight (g/fruit) among tomato cultivars in Gambella Tare and Bako Tibe Districts, Western Ethiopia (Source: own sketch).

Significant genotypic variations were observed for fruit weight (*p* ≤ 0.01) at both locations. Cochoro displayed the highest average weight (146.1 and 148.3 g/fruit), while Komto produced the lowest (45.3 and 48.4 g/fruit) at Bako Tibe and Gambela Tarre, respectively (Tables 5 and 6, Figure 3). Environmental factors likely contribute, along with genetics, to shaping FS. Optimal quality hinges on both varietal potential and a nurturing environment. This aligns with Regassa et al. [27] and Mahmood et al. [43], emphasizing genetic and environmental influences.

#### 3.1.5. PH (cm)

**Figure 4:**
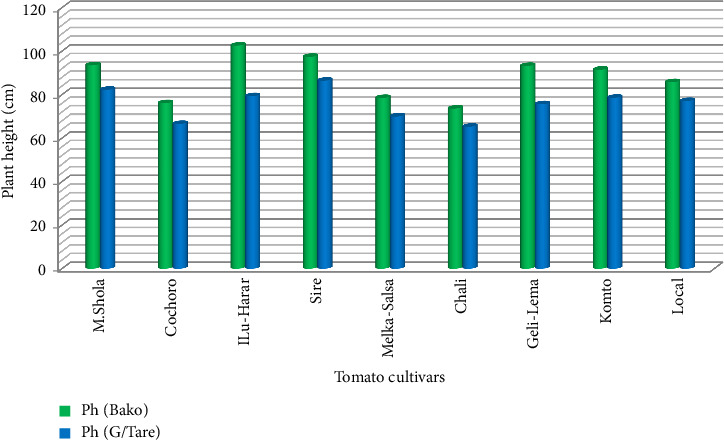
Comparison of plant height (cm) among tomato cultivars in Gambella Tare and Bako Tibe districts, Western Ethiopia (Source: own sketch).

ANOVA revealed highly significant (*p* ≤ 0.01) differences in PH among tomato cultivars at both experimental sites. At Bako sites, ILu-Harar reached the tallest height (102.6 cm), while Chali was the shortest (73.6 cm). At the Gambella Tare site, Variety Chali produced the shortest PH (65.3 cm), while Variety Sire produced the largest PH (86.5 cm) (Tables 5 and 6, Figure 4). Height differences are attributed to varietal genetics. Taller cultivars may require longer growth periods and specific management practices. Short cultivars potentially require less labor and are popular for tropical cultivation. This finding is consistent with earlier studies by Milkinesh and Negash [39] and Begna Bedassa et al. [44] highlighting varietal diversity in PH.

#### 3.1.6. Number of Fruit Clusters per Plant

**Figure 5:**
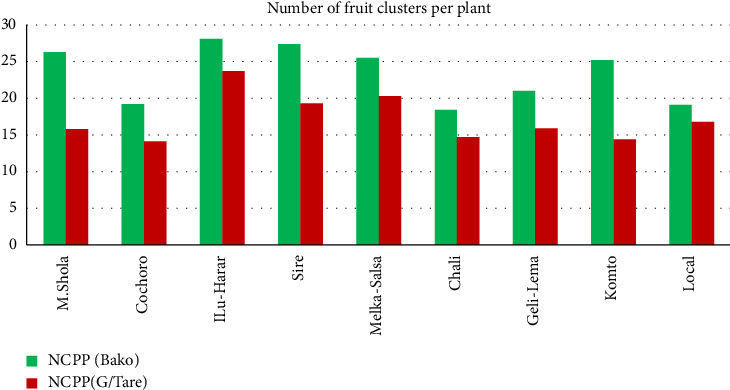
Comparison of number of fruit clusters per plant among tomato cultivars in Gambella Tare and Bako Tibe Districts, Western Ethiopia (Source: own sketch).

Analysis revealed highly significant differences (*p* ≤ 0.01) in cluster number across cultivars at both locations. ILu-Harar produced the highest number of clusters (28.1–23.7) at both locations, while variety Chali generated the lowest clusters (18.4) at Bako and Cochoro (14.1) at Gambella Tare (Tables 5 and 6, Figure 5). Cluster number is potentially linked to plant height and vigorous growth habits. More clusters, along with FS, can influence yield potential [45, 46]. The present study aligns with Begna Bedassa et al.'s [44] showcasing varietal variations in cluster formation.

#### 3.1.7. MFY (t/ha)

**Figure 6:**
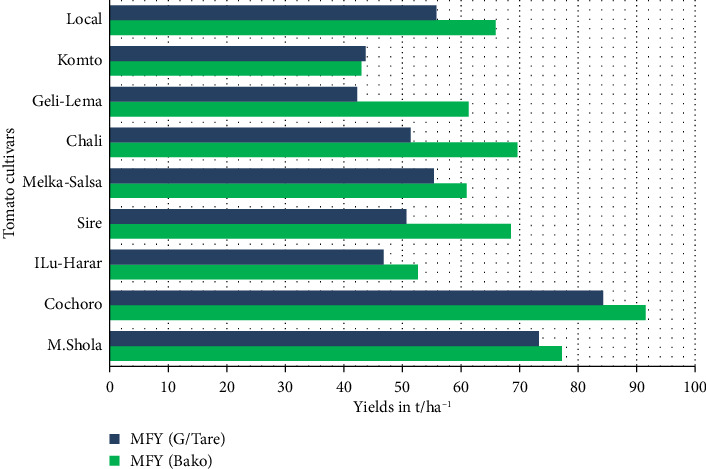
Comparison of marketable fruit yield (t/ha) among tomato cultivars in Gambella Tare and Bako Tibe districts, Western Ethiopia (Source: own sketch).

Significant genotypic variations were observed (*p* ≤ 0.01) at both locations. Cochoro topped yields (91.5 and 84.28 t/ha), while Komto and Geli-Lema had the lowest (43 and 42.24 t/ha) at Bako Tibe and Gambela Tarre, respectively (Tables 5 and 6, Figure 6). Differences are attributed to genetics and agroecological adaptation. Marketable yield is a key for commercialization and farmer income [3]. This aligns with Rida et al. [47] and Chernet et al. [42] reporting yields ranging from 37.1 to 76.2 t/ha.

#### 3.1.8. TFY (t/ha^−1^)

**Figure 7:**
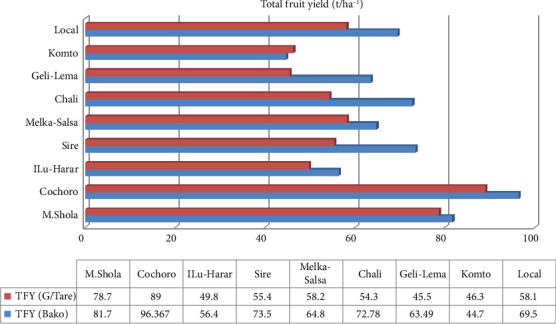
Comparison of total fruit yield (t/ha^−1^) among tomato cultivars in Gambella Tare and Bako Tibe Districts, Western Ethiopia (Source: own sketch).

ANOVA revealed a highly significant (*p* ≤ 0.01) effect of tomato variety on TFY at both locations. Cochoro dominated again (96.37 and 89 t/ha), while Komto and Geli-Lema had the least (44.7 and 45.5 t/ha) at Bako Tibe and Gambela Tarre, respectively (Tables 5 and 6, Figure 7). Environmental factors, plant height, branching, and leaf area are potential contributors to variations. The result falls within the range reported by Getachew and Tewodros [16] and Yeshiwas et al. [48] (86 and 102 t/ha). Also, these findings align with broader literature showcasing variable tomato yields [49].

#### 3.1.9. FS (mm)

Significant genotypic variations were observed (*p* ≤ 0.01) at both locations. Cochoro and Chali varieties had the tallest fruits (61.6 mm and 54.9 mm), whereas Melka-Salsa and Sire varieties produced the shortest fruits (34.2 mm and 36 mm) at Bako Tibe and Gambella Tare locations, respectively (Tables 5 and 6). Size variation is likely due to fruit set, competition, hormones, and nutrient partitioning [50]. The result is consistent with Kumar et al. [51] (40–70 mm range) and deviates from Luitel et al. [52] (41.4–47.7 mm).

#### 3.1.10. FL (cm)

Highly significant genotypic influence (*p* ≤ 0.01) is shown at both locations. The longest FL was recorded from the Sire variety (6.9 cm and 6.5 cm) at Bako Tibe and Gambella Tare, respectively, whereas the shortest FL was recorded from the Komto variety (4.1 cm) at Bako and the ILu-Harar variety (4.2 cm) at the Gambella Tare experimental site; however, values did not differ significantly with other treatments (Tables 5 and 6). In tomatoes, length variations are potentially tied to fruit set, assimilation competition within the plant, and resource allocation [53]. The current finding falls within the range of previous studies [54, 55] (3.91–6.57 cm). However, it contradicts Luitel et al. [52] (3.64–4.23 cm).

#### 3.1.11. FPTH (mm)

ANOVA revealed a highly significant difference among varieties at Bako (*p* ≤ 0.01). Cochoro displayed the maximum pericarp thickness of 6 mm, while the lowest pericarp thickness of 3.4 mm was observed in the Melka-Salsa (Table 5). The observed range aligns with Yesmin et al. [56] and Koutsos and Noutsos [57] (3.12–6.33 mm). The result is also similar to Ashenafi and Demelash's [58] findings of variable pericarp thickness. Slightly lower average thickness compared to Salim et al. [54] (4.40–8.07 mm).

#### 3.1.12. NL per Fruit

A highly significant effect of tomato cultivar on the NL per fruit was observed at the Bako Tibe experimental site (*p* ≤ 0.01). Chali exhibited the fewest locules per fruit (1.2) at Bako Tibe, whereas Melka-Shola possessed the highest number (2.1), albeit not significantly different from Cochoro (2), Sire (1.7), and Komto (1.6) (Table 5). Locule number affects fruit shape and size and is controlled by several quantitative trait loci [59]. The observed minimum NL per fruit aligns with Milkinesh and Negash [39], who reported a minimum of 1.33. However, previous studies by Yesmin et al. [56] and Salim et al. [54] documented higher locule counts, contrasting with the present findings.

### 3.2. Correlation Coefficients

This study evaluated the performance of nine tomato cultivars across two contrasting sites (Bako Tibe and Gambella Tare) to elucidate the complex interplay between genotype, environment, and agronomic traits. Significant variation was observed in DF, PH, number of fruit clusters per plant, FPTH, AFW, size, and MFY (Tables 7 and 8).

Days to 50% of flowering are associated with various plant growth and development patterns, with implications for yield and quality depending on the environment. DF show positive and significant correlations with PH (Bako: *r* = 0.653^∗∗^, Gambella Tare: *r* = 0.375^∗^), NDM (Bako: *r* = 0.608^∗∗^, Gambella Tare: *r* = 0.375^∗^), and number of fruit clusters per plant (Gambella Tare: *r* = 0.532∗), while there are negative and significant correlations: number of fruit sets per plant (Gambella Tare: *r* = −0.510^∗∗^), NPBP (Gambella Tare: *r* = −0.328^∗^), FPC (Gambella Tare: *r* = −0.345^∗∗^) (Tables 7 and 8). This result aligns with Tesfaye et al. [60], Assefa et al. [61], and Boateng et al. [62] findings.

FPTH demonstrates positive correlations with both PH and AFW, suggesting its potential as a marker for improved fruit quality. FPTH correlates positively and significantly with PH (Bako: *r* = 0.658^∗∗^), AFW (Bako: *r* = 0.629^∗∗^, Gambella Tare: *r* = 0.533^∗^), FS (Bako: *r* = 0.555^∗∗^), and MFY (Bako: *r* = 0.465^∗^) (Tables 6 and 7).

AFW emerges as a central parameter, positively linked to yield and other key quality traits, highlighting its importance in breeding programs. AFW showed positive and significant correlations with MFY (Bako: *r* = 0.644^∗∗^, Gambella Tare: *r* = 0.637^∗∗^), TFY (Bako: *r* = 0.634^∗∗^, Gambella Tare: *r* = 0.643^∗∗^), FS (Bako: *r* = 0.892^∗∗^, Gambella Tare: *r* = 0.331^∗^), FL (Bako: *r* = 0.562^∗∗^), and FPTH (Bako: *r* = 0.629^∗∗^, Gambella Tare: *r* = 0.533^∗∗^) (Tables 6 and 7). The current result aligns with Tiwari et al. [63], Yasmeen et al. [64], and Tesfaye et al. [60] findings.

### 3.3. Qualitative Characters

#### 3.3.1. TSS

**Figure 8:**
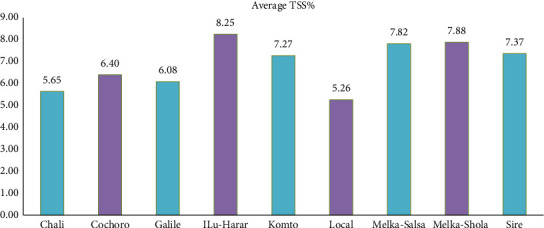
Comparison of mean total soluble solid (TSS) content (%) among tomato cultivars in Gambella Tare and Bako Tibe districts, Western Ethiopia (Source: own sketch).

TSS content, a crucial factor impacting flavor in both processing and fresh market tomatoes, varied significantly among cultivars. ILu-Harar emerged as the frontrunner with 8.25% TSS, followed by Melka-Shola (7.88%) and Melka-Salsa (7.82%). The local variety exhibited the lowest value at 5.26% (Figure 8).

This observed variation may stem from inherent differences in tomato genotypes, environmental factors like temperature, and pre- and postharvest handling practices. This finding aligns with Milkinesh and Negash [39] and Sacco [65], who reported cultivated tomato TSS ranging from 4.0% to 8.5%. Patwary et al. [66] observed similar ranges (4.37%–5.67% in winter and 3.39%–4.77% in summer tomatoes), while Brunele Caliman et al. [67] reported lower values (3.60%–3.83%) under protected conditions. These discrepancies highlight the interplay of genotype, environment, and cultivation practices in determining TSS.

#### 3.3.2. pH Value

**Figure 9:**
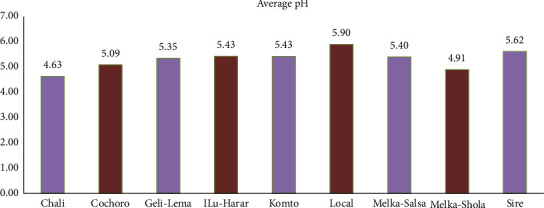
Comparison of mean pH values among tomato cultivars in Gambella Tare and Bako Tibe districts, Western Ethiopia (Source: own sketch).

Alongside TSS, pH significantly affects flavor and sourness. Chali, Melka-Shola, and Cochoro juices displayed a desirable acidic profile, with pH values between 4.63 and 5.09 (Figure 9). These findings resonate with Brunele Caliman et al. [67], who documented the inherent acidity of tomato juices (pH < 7) and its positive correlation with fruit quality.

#### 3.3.3. Ascorbic Acid Content (mg/100)

**Figure 10:**
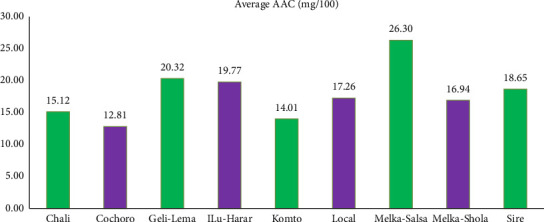
Comparison of mean ascorbic acid concentration (mg/100) among tomato cultivars in Gambella Tare and Bako Tibe districts, Western Ethiopia (Source: own sketch).

Melka-Salsa led the pack in ascorbic acid (vitamin C) content with 26.30 mg/100 g, while Cochoro showed the lowest at 12.81 mg/100 g (Figure 10).

The present observations on the variability in ascorbic acid content (8.55–20.12 mg/100 g) resonate with the findings of Sibi [68] and Dursun et al. [69], who documented similar ranges (13.63–48.22 mg/100 g and 17.33–25.77 mg/100 g, respectively) within tomato cultivars. These studies collectively highlight the substantial influence of cultivar diversity on this essential nutrient, emphasizing the importance of considering this factor when optimizing ascorbic acid content in tomato production [70].

#### 3.3.4. Fruit Density (g/mL)

**Figure 11:**
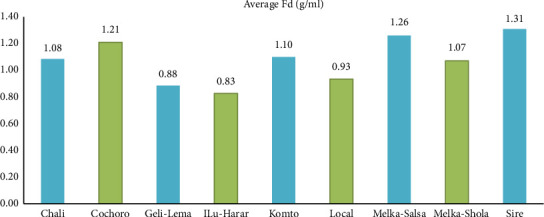
Comparison of mean fruit density (fruits/plant) among tomato cultivars in Gambella Tare and Bako Tibe districts, Western Ethiopia (Source: own sketch).

Fruit density ranged from 0.83 g/mL in ILu-Harar to 1.31 g/mL in Sire, highlighting notable variation among cultivars (Figure 11). This diversity can be attributed to factors like water content, cell wall characteristics, soluble solids concentration, FS, and shape [71]. High fruit density, associated with firmness, juiciness, and shelf life, is often seen as a desirable quality trait for tomatoes [72]. These findings concur with Singh et al. [73], who observed a similar range of fruit density (0.9–1.4 g/mL) across different tomato genotypes.

### 3.4. Overview of Cultivars Performance

#### 3.4.1. Melka-Shola

Melka-Shola showed moderate yields but was noted for its resilience to local pests. Its adaptability to local conditions may explain its consistent performance, as highlighted by Regassa et al. [27], who found that pest-resistant cultivars tend to thrive better in challenging environments.

#### 3.4.2. Cochoro

Cochoro emerged as the highest yielding cultivar, achieving MFY of 91.5 t/ha at Bako and 84.28 t/ha at Gambella Tare. The cultivar's success can be attributed to its superior growth characteristics and adaptability to both locations' microclimates, aligning with findings from Amirahmadi et al. [74], which emphasize the importance of genotype–environment interactions in yield performance.

#### 3.4.3. ILu-Harar

ILu-Harar exhibited good growth but did not match Cochoro's yield levels. Its performance may be influenced by its specific nutrient requirements and sensitivity to environmental stressors, as noted by Gao et al. [75], indicating that certain cultivars require more precise management for optimal yield.

#### 3.4.4. Sire

Sire performed well under specific conditions but showed variability across locations. This variability suggests a need for targeted cultivation practices based on microclimatic conditions, corroborated by research from Šalagovič et al. [76] that highlights the impact of localized environmental factors on cultivar performance.

#### 3.4.5. Melka-Salsa

Melka-Salsa demonstrated superior fruit quality, particularly in ascorbic acid content (26.30 mg/100 g). Its high-quality fruit attributes make it suitable for markets demanding nutritional value, reinforcing findings from Siddiqui et al. [77] that quality traits significantly influence marketability and consumer preference.

#### 3.4.6. Chali

Chali showed average yields but good disease resistance. This resistance may enhance its viability in disease-prone areas, as discussed by Regassa et al. [27], which asserts that disease-resistant cultivars are crucial for sustainable agriculture.

#### 3.4.7. Geli-Lema

Geli-Lema had one of the lowest yields (42.2 t/ha at Gambella Tare). Its underperformance might be linked to poor adaptability to the specific environmental conditions at Gambella Tare, aligning with previous studies that emphasize the significance of local adaptation in agricultural practices.

#### 3.4.8. Komto

Komto also exhibited low yields (43 t/ha at Bako Tibe). The cultivar's poor performance could be due to its inability to cope with the soil conditions or climatic factors at Bako, highlighting the need for careful selection based on environmental compatibility as noted by Amirahmadi et al. [74].

#### 3.4.9. Local Cultivar

The local cultivar performed variably compared to improved varieties. While it may have advantages in terms of local knowledge and adaptability, it often lacks the yield potential of improved cultivars, as supported by findings from Gao et al. [75] regarding the benefits of adopting modern varieties.

### 3.5. Limitations of the Study

The study acknowledges potential biases from selected sites and data collection periods, which may not fully capture seasonal impacts on cultivar performance. Future research should aim to include a broader range of environmental conditions and longer study durations to enhance reliability.

## 4. Economic Implications of Cultivar Choice

### 4.1. Market Value Assessment

The choice of tomato cultivars significantly impacts market value, which is influenced by factors such as yield potential, fruit quality, and consumer preferences. In our study, we found that the cultivar Cochoro consistently yielded the highest MFY across both Bako Tibe (91.5 t/ha) and Gambella Tare (84.28 t/ha). This high yield not only enhances the immediate income potential for farmers but also positions Cochoro favorably in local markets where supply often dictates prices.

Conversely, cultivars like Komto and Geli-Lema, which yielded significantly lower (43 t/ha at Bako Tibe and 42.2 t/ha at Gambella Tare), may struggle to compete in terms of profitability. Lower yields can lead to reduced market presence, making it challenging for farmers to secure favorable prices for their produce. Therefore, selecting high-yielding cultivars is crucial for maximizing market value.

### 4.2. Profitability Analysis in Terms of Market Prices

Fluctuations in market prices based on seasonal availability and consumer demand can significantly affect profitability. Cultivars that mature earlier or have superior quality traits (such as Melka-Salsa, noted for its high ascorbic acid content) may command higher prices during off-peak seasons.

### 4.3. Long-Term Economic Sustainability

Incorporating a broader economic perspective into cultivar selection also aligns with sustainable agricultural practices. By promoting cultivars that are not only high-yielding but also resilient to local climatic conditions and pest pressures, farmers can achieve more consistent production levels over time. This stability is essential for long-term economic sustainability, allowing farmers to invest in their operations and improve their livelihoods.

## 5. Conclusion

This study aimed to address the critical yield gap in tomato (*Solanum lycopersicum* L.) production in Western Ethiopia by evaluating the performance of nine selected cultivars across two contrasting microclimates during the off-season cropping period of 2022–2023. The research was motivated by the pressing need to enhance food security and improve the livelihoods of local farmers, who face significant challenges due to limited access to improved cultivars and inadequate agricultural practices.

The methodology employed a RCBD with three replications, assessing various growth, yield, and quality parameters at two locations: Bako Tibe and Gambella Tare. Statistical analyses, including separate and combined ANOVAs, were conducted using SAS software to evaluate genotype–environment interactions and identify significant differences among cultivars.

Results demonstrated significant genotype–environment interactions for multiple traits, underscoring the importance of microclimatic adaptation in tomato cultivation. Notably, the cultivar Cochoro emerged as the highest yielding option, achieving MFY of 91.5 t/ha at Bako and 84.28 t/ha at Gambella Tare. In contrast, Komto and Geli-Lema yielded significantly less, highlighting the potential for targeted cultivar selection based on local conditions. Additionally, fruit quality varied among cultivars, with Melka-Salsa exhibiting the highest ascorbic acid content.

These findings highlight the critical role of genotype selection in optimizing tomato productivity within specific microclimates, with the strong positive correlation between total MFY and fruit weight per plant underscoring the need for breeding programs to prioritize these traits. The study provides actionable insights into cultivar performance under local off-season conditions while addressing knowledge gaps in identifying tomato varieties suited to Ethiopia's agricultural context. By disseminating this information, producers can make informed decisions to improve yield and fruit quality, thereby strengthening food security and supporting the resilience of Ethiopia's agricultural sector. Future studies should validate these outcomes across multiple growing seasons and broaden germplasm evaluations to incorporate genotypes with enhanced heat and disease tolerance, ensuring adaptability to the country's escalating climatic challenges.

## 6. Future Lines of Work

Future research on tomato cultivation in Western Ethiopia should prioritize a multifaceted approach to address gaps in understanding and practice, with an emphasis on adaptability, sustainability, and resilience. Longitudinal studies across multiple growing seasons and locations are essential to evaluate genotype–environment interactions, particularly how seasonal shifts such as follows:

• Off-season versus main-season planting and microclimate variability influence cultivar performance.
• Identifying stable, high-yielding varieties under fluctuating conditions will require systematic assessments of growth, yield, and quality traits in diverse agroecological settings. Concurrently, optimizing agronomic practices in line with local contexts is critical. This includes developing irrigation strategies aligned with regional rainfall patterns and soil dynamics, such as drip systems to improve water efficiency, and refining nutrient management through comparative studies of organic and synthetic fertilizers to balance productivity with environmental sustainability.
• Integrated pest management strategies must be explored, emphasizing biological control agents to reduce chemical dependency while addressing region-specific pest and disease pressures.
• Parallel efforts in breeding should leverage advanced techniques like marker-assisted selection to map traits linked to drought tolerance, disease resistance, and fruit quality, coupled with cross-breeding programs to merge desirable characteristics from existing cultivars.
• Socioeconomic dimensions warrant equal attention, including market analyses to align varietal development with consumer demand and farmer profitability, alongside targeted education programs to disseminate best practices.
• Finally, proactive climate adaptation research is indispensable. Climate modeling to forecast regional impacts on tomato production, combined with studies on enhancing crop resilience to extreme weather, will equip farmers with strategies to mitigate risks.

## Figures and Tables

**Table 1:** Description of tomato cultivars used in the study.

Varieties	Year of release	Altitude (m.a.s.l)	Growth pattern	Unique characters	Utilization	Maturity days	Research yield (ton/ha)	Seed source
Melka-Shola	1997/8	700–2000	Determinate	Globular fruit shape	Fresh and processing	100–120	—	MARC
Cochoro	2007	800–2000	Semideterminate	Large fruit and green shoulder fruit color before maturity	Fresh and processing	85–90	—	MARC
ILu-Harar	2015	1200–2000	Determinate	Globular fruit shape	Fresh and processing	85–95	—	BARC
Sire	2015	1200–2000	Indeterminate	Round in shape	Fresh and processing	80–90	—	BARC
Melka-Salsa	1998	700–2000	Determinate	Small fruit size and slightly cylindrical	Fresh and processing	100–110	—	MARC
Chali	2007	500–2000	Determinate	Round fruit shape	Fresh and processing	110–120	—	MARC
Geli-Lema	2015	800–2000	Determinate	Red color and oval fruit shape	Fresh	—	—	Axum Greenline PLC & MARC
Komto	2015	1200–2000	Determinate	Perfectly round shape	Fresh and Processing	80–90	—	BARC
Local	—	—	—	—	—	—	—	Bako

*Note:* Source: Adapted from Regassa et al.'s study [27].

**Table 2:** List of quantitative traits and their descriptions.

No	Traits	Code	Description
1	Days to 50% flowering	DF	It was estimated from the date of transplanting until the day 50% of the plants in each plot flowered.
2	Number of days to maturity	NDM	It was obtained from the date of transplanting until 50% of the plants have at least one mature fruit.
3	Plant height (cm)	PH	It was measured using a tape meter (for five randomly selected plants in each plot) by measuring the distance from the base of the plant above the ground to the growing tip, expressed in centimeters.
4	Number of primary branches per plant	NPBP	It was counted at maturity by counting the primary branches on five randomly selected plants in each plot.
5	Number of fruits per plant	NFSP	It was counted by counting the total number of fruits harvested from each plant during successive harvests.
6	Average fruit weight (g)	AFW	This was measured by taking the mean weight of fruit in successive harvests per plant and expressed in grams.
7	Number of clusters per plant	NCP	This was recorded by counting the total number of fruit clusters at maturity.
8	Fruit pericarp thickness (mm)	FPTH	This was measured using vernier calipers on five marketable fruits from each plot, and the average value was recorded [30, 32].
9	Marketable fruit yield per plant (kg)	MFY	It was estimated from fresh weight fruit harvested from five selected plant of each plot and the diseased, deformed, and stressed fruit was removed during weigh.
10	Fruit size (mm)	FS	Fruit size was taken at maturity stage using vernier caliper, and mean values were recorded.
11	Total fruit yield (ton ha^−1^)	TFY	It was determined by recording the total fruit weight (in kilograms) per plot from successive harvests and then converting it to tons per hectare.
12	Fruit length (cm)	FL	It was measured at both the red stages for five marketable fruits from each plot using a vernier caliper, and the average values were recorded.
13	Number of locules	NL	It was taken by counting at least five fruits, and the average values were recorded.
14	Fruits per clusters	FPC	It was counted on at least five plants, and the average values were recorded.
15	Fruit diameter (mm)	Fd	It was measured using vernier calipers on 10 marketable fruits from sample plants in each plot at both the red-ripe and dried stages. The average values were recorded.
16	Stem thickness	STH	It was measured by placing the caliper or ruler perpendicular to the stem and gently but firmly closing it around the stem until it fits snugly.

*Note:* Source: IPGRI [29].

**Table 3:** Combined analysis of variance (ANOVA) for 16 traits of nine tomato cultivars evaluated across two locations (Gambella Tare and Bako), 2022/23.

**Mean squares**								**Pv**		
**Source of variation**	**Replication**	**Location**	**Variety**	**Interaction**	**Error**	**Mean**	**CV**	**Location**	**Variety**	**In/n**
										**Loc ∗ Var**
**d.f**	**2**	**1**	**8**	**8**	**34**					

DF	6.22	647.57	85.38	66.87	14.26	27.94	13.51	< 0.0001	< 0.0001	0.0006^∗∗^
NDM	143.46	60.17	164.69	181.33	22.46	68.98	6.87	0.1109	< 0.0001	< 0.0001^∗∗^
NFSP	278.78	10009.61	507.65	240.90	53.71	34.10	21.49	< 0.0001	< 0.0001	0.0009^∗∗^
NPBP	19.02	74.91	7.41	6.91	7.61	16.88	16.34	0.0035	0.4734	0.522^ns^
AFW	679.96	8.30	6173.77	43.03	257.94	80.08	20.06	0.8587	< 0.0001	0.9939^ns^
Ph	159.74	2008.00	412.81	34.17	22.13	81.84	5.75	< 0.0001	< 0.0001	0.179^ns^
NCPP	7.80	505.27	63.37	13.41	7.35	20.32	13.34	< 0.0001	< 0.0001	0.1064^ns^
MFY	3477.52	1272.16	1095.03	76.43	97.88	60.81	16.27	0.001	< 0.0001	0.6223^ns^
TFY	3862.09	1288.91	1230.75	77.68	109.56	64.39	16.26	0.0016	< 0.0001	0.6816^ns^
FPC	0.77	3.93	0.61	0.71	0.71	3.68	22.85	0.0243	0.553	0.448^ns^
FS	60.56	62.99	250.31	85.12	45.00	44.34	15.13	0.245	0.0002	0.0939^ns^
FD	0.04	0.44	0.17	0.01	0.06	1.08	23.64	0.0138	0.0245	0.988^ns^
FL	0.40	0.66	3.96	0.47	0.29	5.68	9.44	0.1373	< 0.0001	0.1547^ns^
FPTH	0.73	0.01	4.52	0.12	0.64	4.63	17.25	0.9003	< 0.0001	0.9905^ns^
STH	1.23	0.62	1.33	0.10	0.62	8.53	9.21	0.3238	0.0568	0.995^ns^
NL	0.19	0.09	0.41	0.06	0.13	1.55	23.19	0.4123	0.0084	0.8758^ns^

*Note:* DF: days to 50% flowering.

Abbreviations: AFW = average fruit weight (g), CV = coefficient of variation, d.f = degree of freedom, FD = fruit diameter (mm), FL = fruit length (cm), FPC = fruits per cluster, FPTH = fruit pericarp thickness (mm), FS = fruit size (cm), MFY = marketable fruit yield (t/ha^−1^), NCPP = number of cluster per plant, NDM = number of days to maturity, NFSP = number of fruit set per plant, NL = number of locules, NPBP = number of primary branch per plant, ns = nonsignificant, Ph = plant height (cm), STH = stem thickness, and TFY = total fruit yield (t/ha^−1^).

^∗∗^indicates highly significant at 0.01 probability level.

**Table 4:** Combined mean performance of phenological parameters of tomato genotypes evaluated at Gambella Tare and Bako, 2022/23.

Varieties	DF	NDM	NFSP
M. Shola	52.3^a^	75.3^ab^	29.6^de^
Cochoro	48.3^ab^	65.6^d^	30.9^cd^
ILu-Harar	49.8^a^	59.5^e^	38.3^bc^
Sire	49.6^a^	67^cd^	35.2^cd^
Melka-Salsa	43.1^c^	70.5^bcd^	50.6^a^
Chali	42.3^c^	65.8^d^	28.8^de^
Geli-Lema	50.3^a^	71.8^abc^	27.2^de^
Komto	44.0^bc^	68.8^cd^	44.8^ab^
Local	51.8^a^	76.3^a^	21.3^e^

LSD (5%)	4.43	5.56	8.59

*Note:* The means followed by the same letter within the same column are not significantly different at 5% level of significance. DF: days to 50% flowering.

Abbreviations: NDM = number of days to maturity and NFSP = number of fruit sets per plant.

**Table 5:** Mean performance of fruit yield and agronomic traits of tomato genotypes evaluated at Bako, 2022/23.

Varieties	AFW	Ph	NCPP	MFY	TFY	FS	FL	FPTH	NL
M. Shola	62.3^de^	93.5^b^	26.3^a^	77.2^ab^	81.7^ab^	41.4^cd^	5.8^b^	4.1^bc^	2.1^a^
Cochoro	146.1^a^	76.1^d^	19.2^c^	91.5^a^	96.367^a^	61.6^a^	6.4^ab^	6^a^	2^ab^
ILu-Harar	50^e^	102.6^a^	28.1^a^	52.6^cd^	56.4^cd^	42.8^c^	4.3^c^	3.9^bc^	1.5^bc^
Sire	76.7^cd^	97.4^ab^	27.4^a^	68.5^bc^	73.5^bc^	37.7^de^	6.9^a^	4.9^ab^	1.7^abc^
Melka-Salsa	51.4^e^	78.5^d^	25.5^ab^	60.95^bc^	64.8^bc^	34.2^e^	6.2^ab^	3.4^c^	1.3^c^
Chali	102.4^b^	73.6^d^	18.4^c^	69.6^bc^	72.78^bc^	50.7^b^	6^b^	4.5^bc^	1.2^c^
Geli-Lema	87.2^bc^	93.2^b^	21b^c^	61.3^bc^	63.49^c^	48.3^b^	5.7^b^	5.8^a^	1.3^c^
Komto	45.3^e^	91.5^bc^	25.2^ab^	43^d^	44.7^d^	40.2^cd^	4.1^c^	3.7^bc^	1.6^abc^
Local	102.4^b^	85.8^c^	19.1^c^	65.9^bc^	69.5^bc^	51.5^b^	6.3^ab^	4.9^ab^	1.3^c^

LSD at 5%	19.05	6.92	5.07	17.20	17.63	4.33	0.81	1.25	0.54

*Note:* The means followed by the same letter within the same column are not significantly different at 5% level of significance. DF: days to 50% flowering.

Abbreviations: AFW = average fruit weight (g), FL = fruit length (cm), FPTH = fruit pericarp thickness (mm), FS = fruit size (mm), MFY = marketable fruit yield (t/ha^−1^), NCPP = number of cluster per plant, NDM = number of days to maturity, NFSP = number of fruit set per plant, NI = number of inflorescences, NL = number of locules, Ph = plant height (cm), and TFY = total fruit yield (t/ha^−1^).

**Table 6:** Mean performance of fruit yield and agronomic traits of tomato genotypes evaluated at Gambella Tare, 2022/23.

Varieties	AFW	Ph	NCPP	MFY	TFY	FS	FL
M. Shola	67.3^bc^	82.3^ab^	15.8^cd^	73.27^a^	78.7a	39^ab^	5.6^ab^
Cochoro	148.3^a^	66.6^de^	14.1^d^	84.28^a^	89^a^	51^ab^	5.8^ab^
ILu-Harar	51.1^c^	79.3^abc^	23.7^a^	46.78^b^	49.8^b^	41.7^ab^	4.2^c^
Sire	71.3^bc^	86.5^a^	19.3^bc^	50.68^b^	55.4^b^	36^b^	6.5^a^
Melka-Salsa	56.69^c^	70^cde^	20.3^ab^	55.35^b^	58.2^b^	42.9^ab^	6.3^a^
Chali	100.60^b^	65.3^e^	14.7^d^	51.39^b^	54.3^b^	54.9^a^	6.1^a^
Geli-Lema	79.15^bc^	75.6^bcd^	15.9^cd^	42.24^b^	45.5^b^	43.1^b^	5.5^ab^
Komto	48.42^c^	78.6^abc^	14.4^d^	43.7^b^	46.3^b^	44.2^ab^	4.8^bc^
Local	94.19^b^	77.1^abc^	16.8b^cd^	55.8^b^	58.1^b^	36.1^b^	5^bc^

LSD (5%)	33.89	9.55	3.95	16.41	18.1	16.08	1.05

*Note:* The means followed by the same letter within the same column are not significantly different at 5% level of significance. DF: days to 50% flowering.

Abbreviations: AFW = average fruit weight (g), FL = fruit length (cm), FPTH = fruit pericarp thickness (mm), FS = fruit size (mm), MFY = marketable fruit yield (t/ha^−1^), NCPP = number of cluster per plant, NDM = number of days to maturity, NFSP = number of fruit set per plant, NI = number of inflorescences, NL = number of locules, Ph = plant height (cm), and TFY = total fruit yield (t/ha^−1^).

**Table 7:** Pearson's correlation coefficients among 15 quantitative traits in nine tomato genotypes evaluated at Bako, 2022/23.

	DF	NDM	NFSP	NPBP	AFW	Ph	NCP	MFY	TFY	FPC	FS	FL	FPTH	Sth	Fd	NL
DF	1	0.608^∗∗^	−0.101	0.190	−0.289	0.653^∗∗^	0.532^∗^	−0.174	−0.161	0.172	−0.241	−0.214	−0.070	0.216	0.036	0.402^∗^
NDM		1	−0.126	−0.088	−0.320	0.434^∗^	0.233	−0.299	−0.305	0.173	−0.218	−0.163	−0.244	0.141	−0.008	0.140
NFSP			1	0.282	−0.593^∗∗^	−0.090	0.452^∗^	−0.389^∗^	−0.382^∗^	0.345^∗^	−0.568^∗∗^	−0.316	−0.410^∗^	0.267	0.189	−0.167
NPBP				1	−0.439^∗^	0.276	0.568^∗∗^	−0.313	−0.283	0.151	−0.356^∗^	−0.301	−0.369^∗^	0.183	−0.201	0.178
AFW					1	−0.491^∗∗^	−0.667^∗∗^	0.644^∗∗^	0.634^∗∗^	−0.491	0.892^∗∗^	0.562^∗∗^	0.629^∗∗^	−0.181	0.098	0.202
Ph						1	0.484^∗^	−0.269	−0.262	0.354^∗^	−0.389^∗^	−0.346^∗^	−0.262	0.130	−0.128	0.195
NCP							1	−0.375^∗^	−0.352^∗^	0.357^∗∗^	−0.664^∗∗^	−0.338^∗^	−0.457^∗^	0.199	0.276	0.224
MFY								1	0.998^∗∗^	−0.277	0.494^∗∗^	0.573^∗∗^	0.419^∗^	−0.034	0.397^∗^	0.391^∗^
TFY									1	−0.269	0.477^∗^	0.586^∗∗^	0.418^∗^	−0.025	0.393^∗^	0.396^∗^
FPC										1	−0.381^∗^	−0.432^∗^	−0.457^∗^	0.109	−0.015	0.133
FS											1	0.226	0.555^∗∗^	0.018	−0.102	0.178
FL												1	0.382^∗^	−0.156	0.372^∗^	−0.005
FPTH													1	−0.107	−0.164	−0.015
STH														1	0.066	0.053
Fd															1	0.314
NL																1

*Note:* Chxr: character and DF: days to 50% flowering.

Abbreviations: AFW = average fruit weight (g), Fd = fruit diameter (mm), FL = fruit length (cm), FPC = fruits per cluster, FPTH = fruit pericarp thickness (mm), FS = fruit size (cm), MFY = marketable fruit yield (t/ha^−1^), NCPP = number of cluster per plant, NDM = number of days to maturity, NFSP = number of fruit set per plant, NI = number of inflorescences, NL = number of locules, NPBP = number of primary branch per plant, Ph = plant height (cm), STH = stem thickness (mm), and TFY = total fruit yield (t/ha^−1^).

^∗^Indicates a significant correlation (*p* < 0.05).

^∗∗^Signifies a highly significant correlation (*p* < 0.01).

**Table 8:** Pearson's correlation coefficients among 15 quantitative traits in nine tomato genotypes evaluated at Gambella Tare, 2022/23.

	DF	NDM	NFSP	NPBP	AFW	Ph	NCPP	TMFY	FY	FPC	FS	Fd	FL	FPTH	Sth	NL
DF	1	0.375^∗^	−0.510^∗∗^	−0.328^∗^	0.249	0.025	−0.208	0.088	0.102	−0.345^∗^	−0.277	−0.183	−0.114	0.658^∗∗^	−0.103	0.181
NDM		1	−0.463^∗^	0.064	0.222	−0.164	−0.331^∗^	0.069	0.070	−0.383^∗^	−0.019	0.265	0.345	0.115	0.045	−0.057
NFSP			1	−0.00025	−0.079	−0.00037	0.143	0.116	0.120	0.401^∗^	−0.075	0.123	0.262	−0.157	0.140	0.070
NPBP				1	−0.078	0.098	−0.081	−0.276	−0.271	0.198	0.280	0.269	−0.198	−0.316	0.148	−0.029
AFW					1	−0.459^∗^	−0.422^∗^	0.637^∗∗^	0.643^∗∗^	0.153	0.331^∗^	0.259	0.273	0.533^∗∗^	−0.159	0.351^∗^
Ph						1	0.232	−0.242	−0.228	0.048	−0.230	0.037	−0.187	−0.147	0.024	−0.102
NCPP							1	−0.199	−0.196	−0.167	−0.132	−0.080	−0.051	−0.357	0.413	−0.447^∗^
MFY								1	0.998^∗∗^	0.258	0.062	0.109	0.286	0.192	−0.105	0.488^∗∗^
TFY									1	0.252	0.051	0.127	0.296	0.201	−0.098	0.494^∗∗^
FPC										1	0.027	0.048	0.068	−0.070	−0.252	0.251
FS											1	0.090	0.289	−0.079	0.114	−0.049
Fd												1	0.317	0.059	−0.041	0.064
FL													1	0.058	0.021	−0.025
FPTH														1	−0.168	0.149ns
STH															1	−0.390^∗^
NL																1

*Note:* Chxr: character and DF: days to 50% flowering.

Abbreviations: AFW = average fruit weight (g), Fd = fruit diameter (mm), FL = fruit length (cm), FPC = fruits per cluster, FPTH = fruit pericarp thickness (mm), FS = fruit size (cm), MFY = marketable fruit yield (t/ha^−1^), NCPP = number of clusters per plant, NDM = number of days to maturity, NFSP = number of fruits set per plant, NI = number of inflorescences, NL = number of locules, NPBP = number of primary branch per plant, Ph = plant height (cm), STH = stem thickness (mm), and TFY = total fruit yield (t/ha^−1^).

^∗^Indicates a significant correlation (*p* < 0.05).

^∗∗^Signifies a highly significant correlation (*p* < 0.01).

## Data Availability

The raw data and additional information could be made available from the corresponding author upon reasonable request.

## Supporting Information

This supporting information provides detailed data of tomato (*Solanum lycopersicum* L.) traits across multiple microclimates in Western Ethiopia. These tables and figures offer a comprehensive overview of the yield and quality responses of tomato varieties under varying environmental conditions, complementing the main manuscript's findings. Specifically, the supporting information includes detailed tables and figures on the eleven-year (2012–2022) mean rainfall, field temperature, and relative humidity at Bako Tibe, meteorological conditions at Bako Tibe during the 2022 off-cropping season, decadal weather trends at the Gambella Tarre Experimental Site, decadal rainfall and rainy day patterns at the Gambella Tarre Experimental Site, diversity of the studied tomato leaf morphology, and laboratory assessment of tomato quality parameters: ascorbic acid content (%), total soluble solids (%), fruit weight, and pH, and a visual compendium of field activities, encompassing nursery site preparation to tomato crop stand establishment. Appendix Table 1: Eleven-year (2012–2022) mean rainfall, field temperature, and relative humidity at Bako Tibe. Appendix Table 2: Meteorological conditions at Bako Tibe during the 2022 off-cropping season. Appendix Figure 1: Decadal weather trends at the Gambella Tarre Experimental Site, Oromia, Ethiopia. Appendix Figure 2: Decadal rainfall and rainy day patterns at the Gambella Tarre Experimental Site, Oromia, Ethiopia. Appendix Figure 3: Diversity of tomato leaf morphology. Appendix Figure 4: Laboratory assessment of tomato quality parameters: ascorbic acid content (%), total soluble solids (%), fruit weight, and pH.
