# Supplementary material for: Tailoring Tomato (Solanum lycopersicum L.) Traits to Microclimates: A Multilocation Evaluation of Yield and Quality Responses in Western Ethiopia
Source: Scientifica (Cairo). 2025 Apr 28;2025:6345142. doi: 10.1155/sci5/6345142 (PMC12052464; doi:10.1155/sci5/6345142)
Supplement: Supporting Information — Additional supporting information can be found online in the Supporting Information section. [file 6345142.f1.docx]

**APPENDICES (Supplementary Material)**

Appendix Table 1: Eleven-Year (2012-2022) Mean Rainfall, Field Temperature, and Relative Humidity at Bako Tibe

| **Year** | **RF (mm)** | **Temperature ^o^C** |  | **RH%** |  |
| --- | --- | --- | --- | --- | --- |
|  |  | **Minimum** | **Maximum** |  |  |
|  |  |  |  |  |  |
| 2012 | 886.50 | 13.6 | 28.8 | 57.83 |  |
| 2013 | 1431.10 | 12.8 | 28.9 | 49.80 |  |
| 2014 | 1067.10 | 13.8 | 28.5 | 48.40 |  |
| 2015 | 944.33 | 12.3 | 29.8 | 49.33 |  |
| 2016 | 1316.74 | 13.9 | 29.7 | 50.20 |  |
| 2017 | 1598.00 | 12.8 | 29.0 | 51.00 |  |
| 2018 | 1161.70 | 14.0 | 29.8 | 49.90 |  |
| 2019 | 1332.30 | 13.7 | 28.7 | 52.30 |  |
| 2020 | 1605.10 | 14.4 | 29.3 | 52.30 |  |
| 2021 | 1371.30 | 14.0 | 27.9 | 50.80 |  |
| 2022 | 1735.10 | 14.0 | 28.1 | 53.50 |  |
| **Mean** | **1313.57** | **13.57** | **28.95** | **51.40** |  |

Appendix Table 2: Meteorological Conditions at Bako Tibe during the 2022 Off-Cropping Season

| Weather | Jan. | Feb. | Mar. | Apr. | May. | Jun. | Jul. | Aug. | Sept. | Oct. | Nov. | Dec. | Total | mean |
| --- | --- | --- | --- | --- | --- | --- | --- | --- | --- | --- | --- | --- | --- | --- |
| RF | 28.70 | 10.20 | 42.60 | 118.00 | 190.90 | 304.90 | 369.40 | 328.80 | 193.90 | 122.10 | 6.40 | 19.20 | 1735.10 | --- |
| Min | 13.9 | 14.3 | 13.7 | 13.5 | 14 | 14 | 14.7 | 14.3 | 14.2 | 14.3 | 13.3 | 13.5 | 167.70 | 14.0 |
| Max | 30.4 | 31.4 | 29.2 | 27.9 | 27.3 | 26.9 | 24.5 | 25.5 | 27.4 | 27.9 | 29.1 | 29.5 | 337.00 | 28.1 |
| RH% | 53.00 | 51.00 | 54.00 | 55.00 | 57.00 | 56.00 | 54.00 | 54.00 | 53.00 | 52.00 | 50.00 | 53.00 | 642.00 | 53.50 |

Appendix Figure 1: Decadal Weather Trends at the Gambella Tarre Experimental Site, Oromia, Ethiopia

Appendix figure 2: Decadal Rainfall and Rainy Day Patterns at the Gambella Tarre Experimental Site, Oromia, Ethiopia

Appendix Figure 3: Diversity of Tomato Leaf Morphology.

Appendix Figure 4: Laboratory Assessment of Tomato Quality Parameters: Ascorbic Acid Content (%), Total Soluble Solids (%), Fruit Weight, and pH.

Appendix Figure 5: A Visual Compendium of Field Activities, Encompassing Nursery Site Preparation to Tomato Crop Stand Establishment.
